# Specific Residues in the 2009 H1N1 Swine-Origin Influenza Matrix Protein Influence Virion Morphology and Efficiency of Viral Spread *In Vitro*

**DOI:** 10.1371/journal.pone.0050595

**Published:** 2012-11-27

**Authors:** Kristy M. Bialas, Emily A. Desmet, Toru Takimoto

**Affiliations:** Department of Microbiology and Immunology, University of Rochester Medical Center, Rochester, New York, United States of America; The University of Hong Kong, China

## Abstract

In April 2009, a novel influenza virus emerged as a result of genetic reassortment between two pre-existing swine strains. This highly contagious H1N1 recombinant (pH1N1) contains the same genomic background as North American triple reassortant (TR) viruses except for the NA and M segments which were acquired from the Eurasian swine lineage. Yet, despite their high degree of genetic similarity, we found the morphology of virions produced by the pH1N1 isolate, A/California/04/09 (ACal-04/09), to be predominantly spherical by immunufluorescence and electron microscopy analysis in human lung and swine kidney epithelial cells, whereas TR strains were observed to be mostly filamentous. In addition, nine clinical pH1N1 samples collected from nasal swab specimens showed similar spherical morphology as the ACal-04/09 strain. Sequence analysis between TR and pH1N1 viruses revealed four amino acid differences in the viral matrix protein (M1), a known determinant of influenza morphology, at positions 30, 142, 207, and 209. To test the role of these amino acids in virus morphology, we rescued mutant pH1N1 viruses in which each of the four M1 residues were replaced with the corresponding TR residue. pH1N1 containing substitutions at positions 30, 207 and 209 exhibited a switch to filamentous morphology, indicating a role for these residues in virion morphology. Substitutions at these residues resulted in lower viral titers, reduced growth kinetics, and small plaque phenotypes compared to wild-type, suggesting a correlation between influenza morphology and efficient cell-to-cell spread *in vitro*. Furthermore, we observed efficient virus-like particle production from cells expressing wild-type pH1N1 M1, but not M1 containing substitutions at positions 30, 207, and 209, or M1 from other strains. These data suggest a direct role for pH1N1 specific M1 residues in the production and release of spherical progeny, which may contribute to the rapid spread of the pandemic virus.

## Introduction

Influenza viruses belong to the *orthomyxoviridae* family consisting of negative sense, single-stranded, RNA viruses with segmented genomes [1]. Even with the availability of annual vaccines against human influenza infection, seasonal epidemics still result in nearly 500,000 deaths worldwide [2]. Additionally, influenza has been the cause of several global pandemics, most notably the 1918 Spanish flu which killed between 20 to 50 million people, and more recently the 2009 H1N1 swine-origin outbreak (pH1N1) [3]. This novel pH1N1 virus that emerged in April 2009, was declared a pandemic just two months later, already having spread to more than 70 countries. Sequence analysis of its gene segments revealed that several reassortment steps led to the emergence of this virus, as it contains HA, NP, and NS genes from the classical swine lineage, NA and M from the Eurasian swine lineage, a human PB1 that was seeded from an avian virus in approximately 1968, and avian PA and PB2 genes [4]. Six of the eight pH1N1 gene segments are shared with North American triple reassortant (TR) swine viruses that, despite their longtime establishment in the swine population, rarely infect humans and exhibit limited human-to-human transmission [5], [6], [7]. Because the two remaining gene segments, NA and M, were acquired from the Eurasian swine lineage, it is possible that these Eurasian swine genes and/or additional mutations created after the reassortment, contribute to the enhanced transmission of the pH1N1 virus among humans.

Both the NA and M segments encode viral proteins with key roles in the influenza assembly, budding and release processes, which are required for efficient viral transmission. NA encodes the type II transmembrane protein, neuraminidase, which is found as a tetramer on the surface of the host-derived lipid envelope. It functions during the final stage of viral budding where it cleaves sialic acid containing receptors to allow for the release of progeny virions from infected cell surfaces [8]. A recent study investigating the contribution of the Eurasian-origin NA and M segments to the enhanced spread of the pH1N1 strain reported a direct role for the novel NA protein in efficient transmission between ferrets [9]. In this report, all naïve animals exposed to ferrets which had been infected intranasally with the pH1N1 isolate, A/California/07/09 (Rec pH1N1), tested positive for influenza by neutralization assays, indicating 100% transmission efficiency. However, upon replacement of Rec pH1N1 NA and M with segments derived from the TR lineage, transmission efficiency was reduced to 50%, similar to that observed by wild-type TR and Eurasian viruses. This observation suggests an important role for at least one of the Eurasian segments in enhanced viral transmission of Rec pH1N1. Furthermore, in this same study, NA originating from the Eurasian lineage was shown to exhibit higher neuraminidase activity *in vitro* compared to NA with TR origin. Moreover, strains containing Eurasian origin NA with increased enzymatic activity released more viral particles, thereby proposing a mechanism by which the NA segment may be contributing to enhanced viral transmission of the pH1N1 virus.

The M segment of influenza encodes two viral proteins, matrix protein (M1) and the M2 ion channel. While M2 is essential for uncoating of the virus during entry, M1 is known to be the key component for both assembly and budding [8]. Accumulation of M1 at the plasma membrane, and its interaction with the cytoplasmic tails of the viral surface proteins, are thought to initiate bud formation by inducing membrane curvature [10], [11], [12]. Additionally, M1 interaction with both newly synthesized viral ribonucleoproteins (vRNPs) and the viral nuclear export protein (NEP) are known to be required for translocation of the viral genome from the host cell nucleus [13], [14], [15], [16], [17], [18], [19]. These essential functions of M1 during the production of progeny virions support a role for the novel M segment in the enhanced transmission of the pH1N1 strain. Strengthening this hypothesis, Chou et al. recently showed that A/Puerto/Rico/8/34 (PR8) expressing the M segment from A/California/04/2009 (ACal-04/09) has increased transmission efficiency *in vivo* using the guinea pig model [20]. In this study, no viral transmission occurred between guinea pigs infected with wild-type PR8 and naïve animals, whereas 50% transmission efficiency was observed between guinea pigs infected with the PR8 recombinant expressing ACal-04/09 M. Though these data implicate a role for the pH1N1 M segment, specifically the M1 protein, in the spread of influenza, the exact mechanism and key residue(s) that determine efficient transmission remain unknown.

Specific M1 residues are also known to affect virion morphology. Influenza virions can range from 100 nm spheres to filamentous particles reaching several micrometers in length [21]. Evidence for M1 influence on virion morphology was first provided in a study which showed that replacement of the M gene of A/WSN/33, a spherical producing strain, with that of the filamentous virus A/Udorn/72 resulted in filamentous virus morphology [22]. Specific amino acids within the M1 protein were later found to be required for the production of filamentous particles, including residues 41, 95, 102, 204, and 218 [22], [23], [24]. Though it is known that influenza morphology affects virus production, its role in viral transmission is still unclear. Early reports showed that most influenza strains isolated from humans are predominantly filamentous and, upon continual passage in egg or tissue culture, adopt a more uniformly spherical morphology [25], [26]. This switch in morphology correlates with an increase in virus titer [26], [27]. Therefore, it is likely that the increased levels of virus production by spherical influenza strains results in more efficient viral transmission.

In this study, we characterized the morphology of pH1N1 isolates, and found that they are predominantly spherical in our cultured cells. This was in contrast to the filamentous phenotype we observed with the closely related, but poorly transmissible, TR swine viruses. In addition, we found that, unlike other strains, pH1N1 M1 by itself can efficiently induce virus-like particles from transfected cells. To evaluate the contribution of M1 mutations in virus morphology, we rescued various pH1N1 virus containing mutations at M1 residues different between pH1N1 and TR. Our results revealed that pH1N1 M1 residues 30, 207 and 209 are involved in regulation of virus morphology and enhanced viral spread *in vitro*. These data suggest that a few mutations present in pH1N1 contribute to morphological change and efficient transmission of influenza viruses.

## Results

### Virion Morphology of the 2009 H1N1 Swine-origin Influenza Viruses

**Figure 1:**
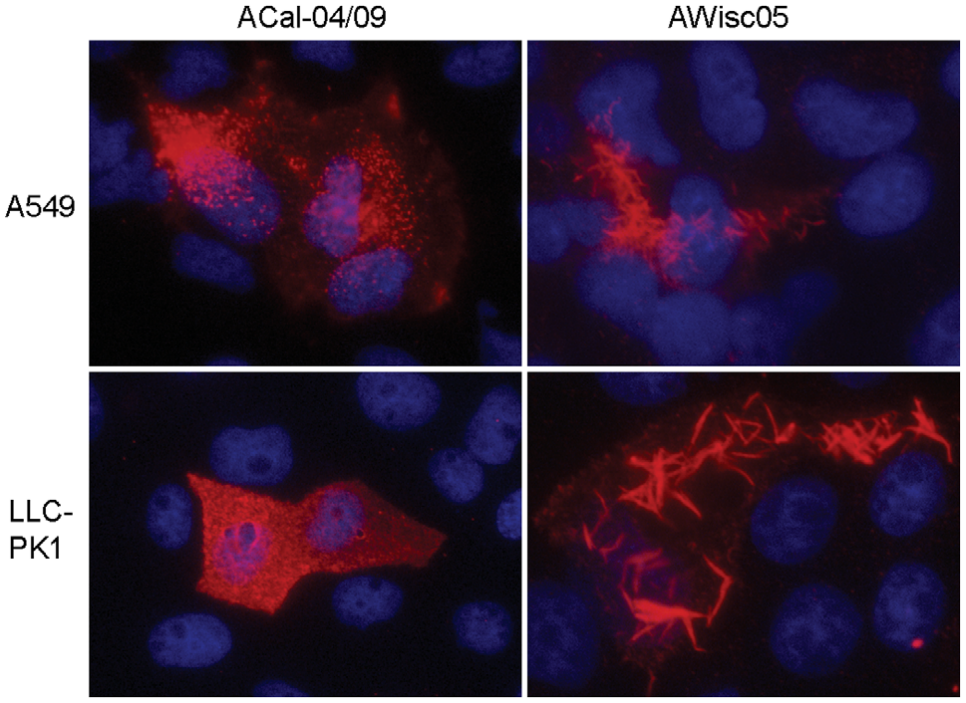
TR swine virus but not the pH1N1 strain induces filament formation from infected human lung and swine kidney cells. Human A549 and swine LLC-PK1 cells infected with the ACal-04/09 or AWisc05 were processed for visualization of viral surface proteins 18 hpi by IF microscopy using anti-H1N1 mouse serum followed by anti-mouse IgG-Texas Red.

Human infection with TR swine viruses prior to 2009 was a rare incidence, and mostly self limiting. Conversely, pH1N1, while sharing most of the same gene segments with TR swine viruses, transmitted readily from person-to-person and hence began the first influenza pandemic of the 21^st^ century. Although multiple factors are likely to be involved in the emergence of the pH1N1, efficient virion production is among the requirements for the outbreak. Because efficient virion production has previously been shown to correlate with spherical rather than filamentous particle formation, we first determined the morphology of pH1N1 and TR viruses. Initially, we investigated the presence or absence of filamentous protrusions at the surface of human lung epithelial A549 cells infected with the viruses by immunufluorescence (IF) analysis. A549 cells were infected with either the TR strain A/Wisconsin/87/2005 (H1N1, AWisc05), or a pH1N1 virus A/California/04/2009 (ACal-04/09). At 18 h, the cells were fixed and processed for visualization of viral surface proteins using anti-H1N1 mouse serum. Noticeably, cells infected with AWisc05 exhibited long filamentous structures on their surface (Fig. 1). This phenotype was not unique to AWisc05, as four additional TR isolates tested, A/Iowa/01/2006 (H1N1), A/Iowa/02/2006 (H1N1), A/Ohio/02/2007 (H1N1) and A/Minnesota/03/2008 (H1N1) were also found to induce filament formation (data not shown). In contrast to TR viruses, infection with ACal-04/09 resulted in a staining pattern consisting of small punctae covering the entire cell surface that is typically seen by spherical influenza strains. Morphological differences between AWisc05 and ACal-04/09 were also observed in swine kidney fibroblast LLC-PK1 cells, indicating that virion morphology is conserved between these cell types (Fig. 1).

**Figure 2:**
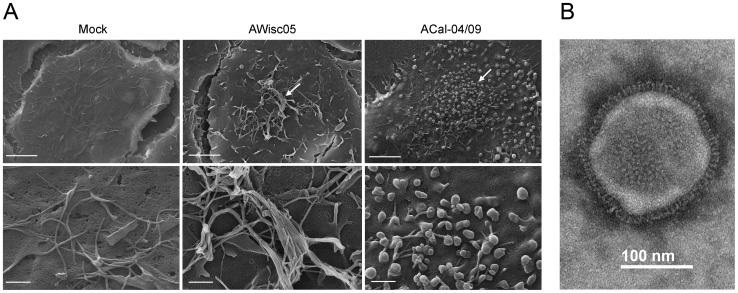
Electron microscopy analysis of TR or pH1N1-infected A549 cells. (**A**) Mock infected A549 cells, and A549 cells infected with AWisc05 or ACal-04/09 were fixed at 18 h and processed for scanning electron microscopy. Bars measure 10 µm (top) and 1 µm (bottom). Arrows point to virions budding from infected cell surfaces. (**B**) Electron micrograph of negatively stained ACal-04/09 virion released from infected A549 cells.

We further examined the structure of budding virions by scanning electron microscopy. Using this method, we observed a similar induction of filaments in the AWisc05-infected A549 cells as was seen by IF analysis. In contrast, the presence of small spherical structures was detected on the surface of cells infected with the ACal-04/09 virus (Fig. 2A). We also characterized the morphology of ACal-04/09 virions released from infected A549 cells by transmission electron microscopy. In order to avoid distortion of particle shape, culture supernatants were harvested after 24 h and concentrated by low speed centrifugation through a filter unit and absorbed onto carbon-coated grids. The diameters of 50 negatively stained virions were measured, after which they were characterized as either spherical or filamentous. From this data, we found more than 98% to be spherical, defined as any particle having a length that is less than twice its width (Table 1). The electron micrograph in Figure 2B represents the majority of ACal-04/09 virions. Collectively, these data suggest ACal-04/09 as a predominantly spherical virion producing strain.

**Table 1:** Measurement of ACal-04/09 viral particles.

Length/width ratio	# of particles
1.0	9
1.1	12
1.2	14
1.3	5
1.4	3
1.5	4
1.6	1
1.7	1
1.8	0
1.9	0
2.0	0
>2.0	1

**Figure 3:**
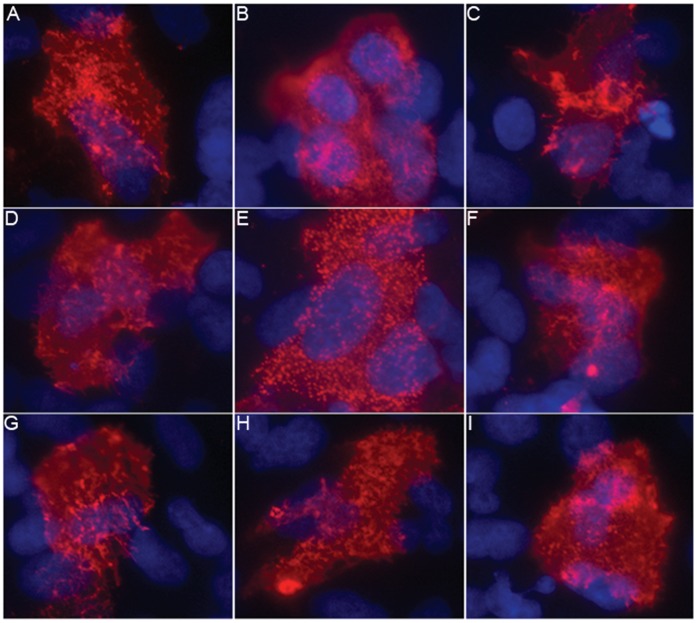
Direct infection of A549 cells with clinical pH1N1 nasal swabs does not induce long filaments. (A–I) A549 cells were directly infected with nasal swab specimens collected from nine patients with confirmed pH1N1 infection. At 18 h, cells were processed for surface staining of viral proteins as described for Fig. 1.

Because our data is in disagreement with previously published work [3], [9], [28], [29], we characterized additional pH1N1 isolates by IF microscopy. In this experiment, A549 cells were infected with nasal swab specimens obtained from patients with confirmed pH1N1 infection, which had no prior passage history in egg or tissue culture. While we did observe some variation between the isolates, most appeared to exhibit a similar punctae staining pattern as ACal-04/09 suggesting that the pH1N1 strains, in general, produce spherical virions in human lung cells (Fig. 3). Further characterization of the clinical isolates in MDCK cells yielded comparable results (data not shown), suggesting that viral, rather than cellular factors are playing a central role in the determination of virion morphology. Additionally, sequence analysis of the M1 proteins from each of the isolates was performed, revealing no amino acid differences between clinical samples and the ACal-04/09 virus, making ACal-04/09 a suitable strain for further study.

### Specific Residues in the pH1N1 M1 Protein are Required for Spherical Virion Morphology

A variety of influenza viral proteins have been shown to affect virion morphology including each of the transmembrane proteins, the nucleoprotein, and M1 [22], [23], [24], [30], [31], [32], [33], [34], [35]. Because pH1N1 acquired the M gene from a Eurasian swine virus, we hypothesized that the difference in virion morphology between TR and pH1N1 was due to variations in the M1 protein sequence. To test this, we compared 153 classical swine and 130 Eurasian swine M1 sequences collected between the years of 2000 and 2008 with ACal-04/09 M1 (Table 2). From this analysis, we identified four amino acid differences between TR and ACal-04/09 M1 proteins at positions 30, 142, 207, and 209. Two of these residues, 142 and 209, were shared between Eurasian swine and pH1N1 viruses, and are conserved among members of the Eurasian swine lineage. pH1N1 M1 residues Ser at 30 and Asn at 207 were rarely found in swine isolates. Moreover, extensive analysis of all available pH1N1 M1 sequences revealed each of these residues to be highly maintained throughout the 2009, 2010, and 2011 seasons.

**Table 2:** Amino acid differences between classical swine, Eurasian swine and the 2009 pH1N1 M1 proteins.

	M1 residue			
	30	142	207	209
Classical Swine	**D** (153/153)	**V** (152/153)G (1/153)	**S** (148/153)G (3/153)N (2/153)	**A** (153/153)
Eurasian Swine	G (126/130)S (4/130)	A (130/130)	S (119/130)N (8/130)G (1/130)	T (130/130)
pH1N1(human 2009 isolates)	**S** (3,780/4,162)D (334/4,162)N (46/4,162)G (2/4,162)	**A** (3,825/4,162)S (330/4,162)V (6/4,162)Xaa (1/4,162)	**N** (3,830/4,162)S (329/4,162)others (3/4,162)	**T** (3,828/4,162)A (333/4,162)Xaa (1/4,162)
pH1N1(human 2010–2012 isolates)	**S** (699/721)N (16/721)others (6/721)	**A** (720/721)G (1/721)	**N** (721/721)	**T** (721/721)

Number of isolates containing the indicated residues/total number of isolates is indicated.

**Figure 4:**
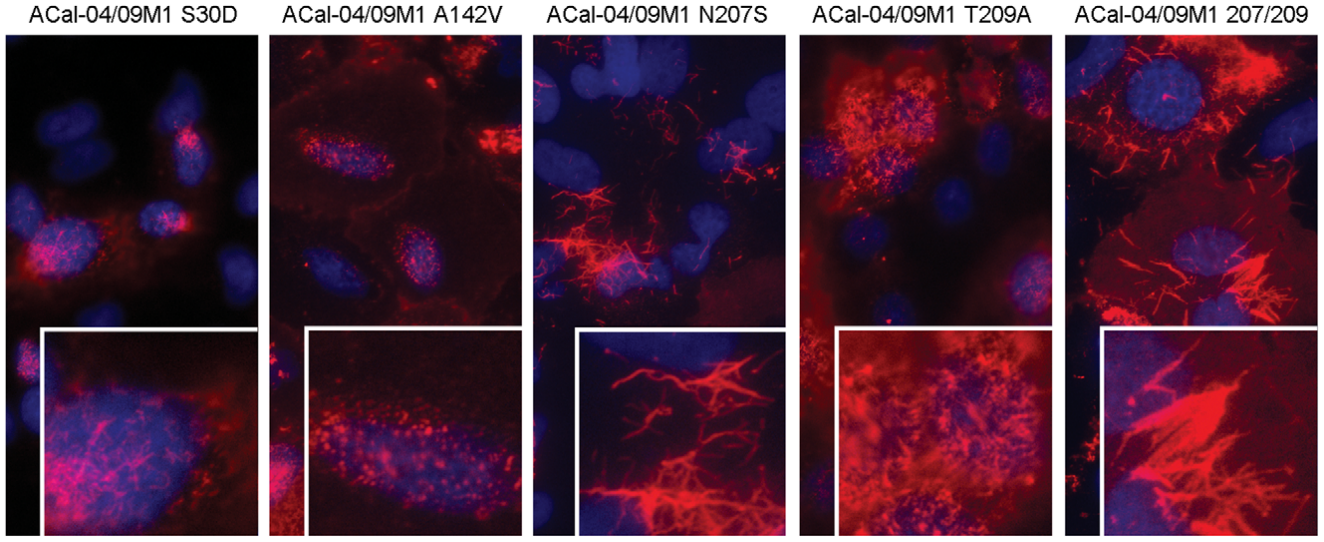
Substitutions at positions 30, 207 and 209 in the ACal-04/09 M1 protein resulted in filament formation in infected cells. A549 cells were infected with ACal-04/09 viruses containing single or multiple substitutions at positions 30, 142, 207, and 209 in the M1 protein as indicated. Cells were fixed and processed for visualization of viral surface proteins 18 h by IF microscopy as described for Fig. 1.

To determine if these M1 residues affect the morphology of pH1N1, we rescued ACal-04/09 viruses in which one or more of the pandemic M1 residues were substituted with the corresponding TR swine residues (Table 3). Rescued viruses were plaque cloned and stock viruses were prepared in MDCK cells. M genes of the rescued viruses were sequenced and confirmed to contain only the designed mutations. First, we characterized the morphology of budding virions by IF in A549 cells. Replacement of residue 142 had no affect on virion morphology, resulting in virus with a similar punctae staining pattern as ACal-04/09 (Fig. 4). Single substitution of residue 30, or 209 slightly altered virion morphology, producing short filaments in infected cells. In sharp contrast, replacement of 207 or both 207 and 209 to those of TR residues, caused an induction of filamentous structures resembling those of AWisc05 (Fig. 4). Consistent results were obtained in primary human lung epithelium (data not shown). These data suggest that residues 30S, 207N and 209T in pH1N1 M1 play a key role in spherical virion formation.

**Table 3:** Virus titer and plaque phenotypes of recombinant ACal-04/09 viruses.

Viruses	Residues 30/142/207/209	Maximum Titer(LogTCID_50_/ml)	PlaqueSize (mm)
**A/California/04/2009 WT**	S/A/N/T	7.4	0.7
**A/California/04/2009 M1 S30D**	D/A/N/T	6.9	0.3
**A/California/04/2009 M1 A142T**	S/V/N/T	6.6	0.5
**A/California/04/2009 M1 N207S**	S/A/S/T	6.0	0.3
**A/California/04/2009 M1 T209A**	S/A/N/A	6.6	0.4
**A/California/04/2009 M1 207/209**	S/A/S/A	6.0	0.3

### Morphological Change Correlates with Efficiency of Viral Spread *in vitro*

**Figure 5:**
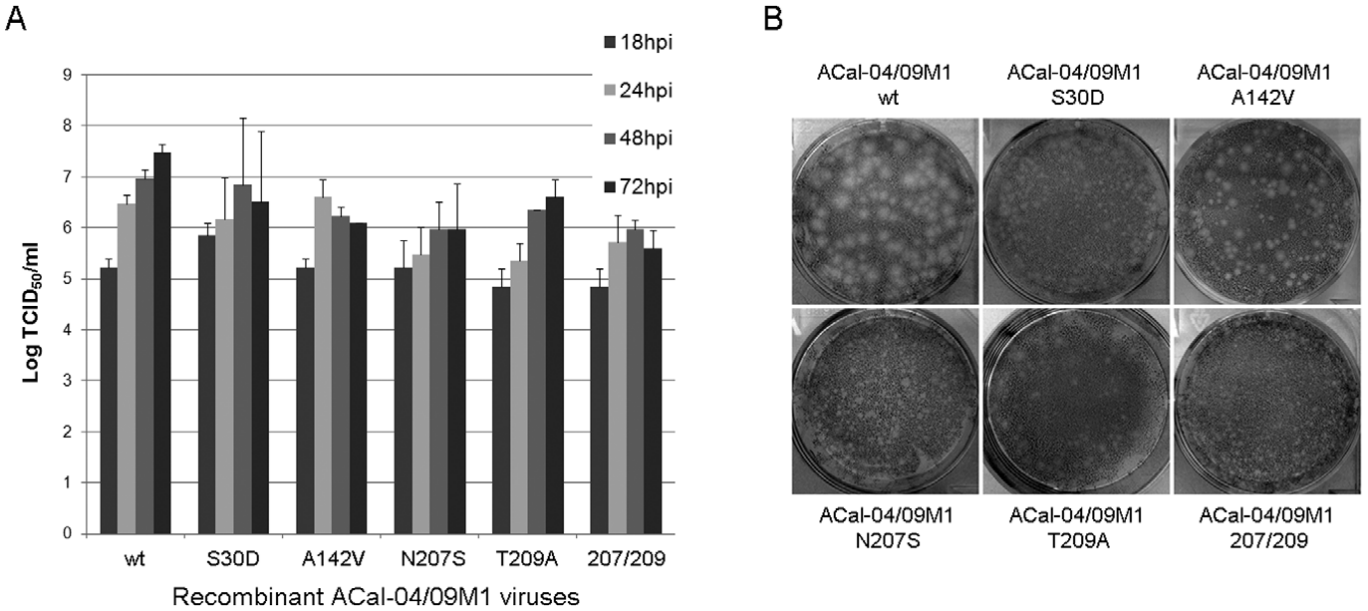
Substitutions at positions 30, 207 and 209 in the ACal-04/09 M1 protein reduce viral growth and spread *in vitro*. (**A**) Growth kinetics of WT and mutant ACal-04/09 viruses were measured in MDCK cells infected at MOI 0.01. Virus titer was determined by TCID_50_ at the indicated time points. Error bars signify standard deviation. (**B**) Plaque phenotypes of WT and recombinant ACal-04/09 viruses were characterized in MDCK cells 4 dpi following crystal violet staining.

Next, we examined the growth rates and plaque size of the wild-type and mutant viruses to determine efficiency of viral spread *in vitro*. Data from our multi-cycle infection showed that single replacement of residue 30, 142, 207, or 209 in the ACal-04/09 M1 protein reduced overall viral production as well as growth kinetics albeit to different extents (Fig. 5A). ACal-04/09 M1 A142V virus, which retained spherical virion morphology, grows as fast as ACal-04/09 WT until 24 h. In contrast, growth rate of viruses containing substitutions at position 207 or both 207 and 209 was slowest and the maximum virus titers of the viruses were 25 times less than that of WT (Table 3). Coinciding with our multi-cycle growth curve analysis, the plaque size of ACal-04/09 M1 A142V following 4 day incubation was only slightly reduced compared to wild-type (Fig. 5B). Of the remaining viruses, those with substitutions at residues 207 and 209 formed barely visible plaques.

### Expression of the ACal-04/09 M1 Protein is Sufficient for Virus-like Particle Production

**Figure 6:**
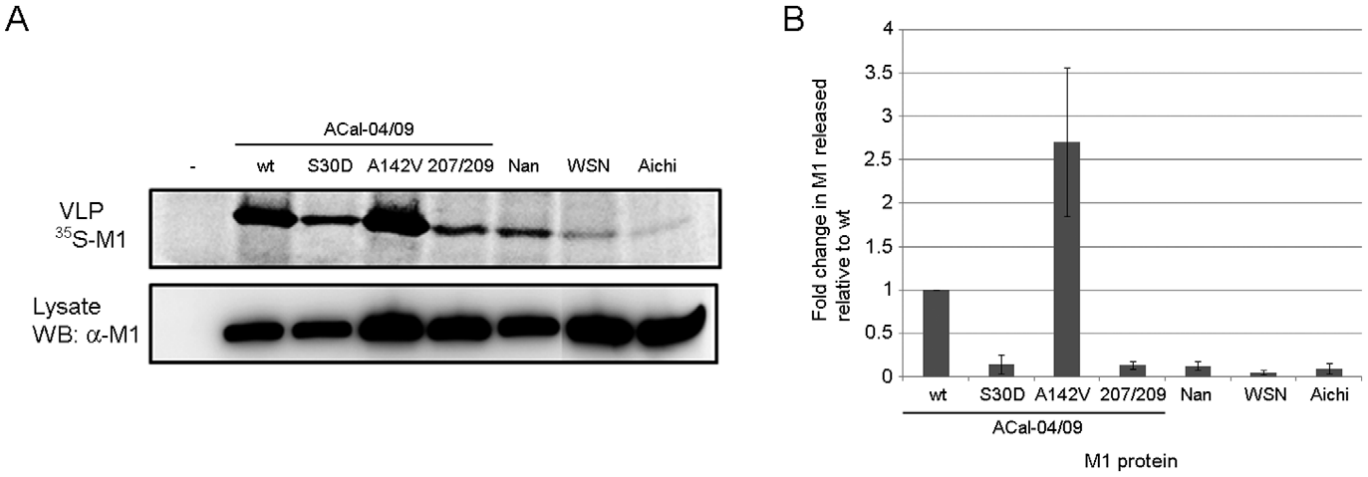
Mutations of ACal-04/09 M1 at residues 30 or 207 and 209 reduced VLP production from transfected cells. (A) 293T cells were transfected with expression plasmids containing WT or mutant ACal-04/09, or other M1 genes. Purified radiolabeled VLPs in culture supernatants were analyzed by SDS-PAGE and total M1 in cell lysates was determined by Western blot analysis. (B) Band intensities were quantified using BioRad software. VLP production is shown as the amount of M1 released into cell culture supernatant normalized to M1 produced in cell lysates. ACal-04/09 WT was set to 1. Data shown are an average of three individual experiments. Error bars represent standard deviation.

Data described above suggest a direct role for the pH1N1 M1 protein, specifically residues 30, 207 and 209, in determining influenza morphology, enhancing viral production, and mediating efficient viral spread *in vitro.* To explore the possible mechanism by which the pH1N1 M1 protein contributes to efficient release of highly transmissible virions, we further characterized the ACal-04/09 M1 for its ability to induce virus-like particles (VLPs) by itself in cultured cells. Previous studies have shown that expression of influenza NA, M2, or HA alone, but not M1, is sufficient for efficient VLP production [36], [37], [38]. We quantified VLP production from ^35^S-labeled 293T cells transfected with plasmids expressing wild-type or mutant ACal-04/09 M1, or with plasmids expressing avian or human virus M1. In agreement with previous reports, we found trace amounts of M1 protein released into the supernatant of 293T cells transfected with A/chicken/Nanchang/3-120/01 (H3N2, Nan), A/WSN/33 (H1N1, WSN), and A/Aichi/2/68 (H3N2, Aichi) M1 plasmids (Fig. 6A). However, cells expressing ACal-04/09 M1 induced high levels of VLP production. Release of ACal-04/09 M1 was 7.7-, 18.9-, and 10.3-times more efficient than that of Nan, WSN and Aichi, respectively. Interestingly, VLP production was reduced by 76% with S30A mutation, and 87% with N207S plus T209A mutations (Fig. 6B). In contrast, the A142V mutation, which did not affect virus morphology, showed no reduction in VLP production, but even more efficiently produced VLP from transfected cells. These results indicate a unique feature of the pH1N1 M1 protein to induce and complete budding at the plasma membrane by itself, which was not observed with other influenza virus M1 protein tested.

**Figure 7:**
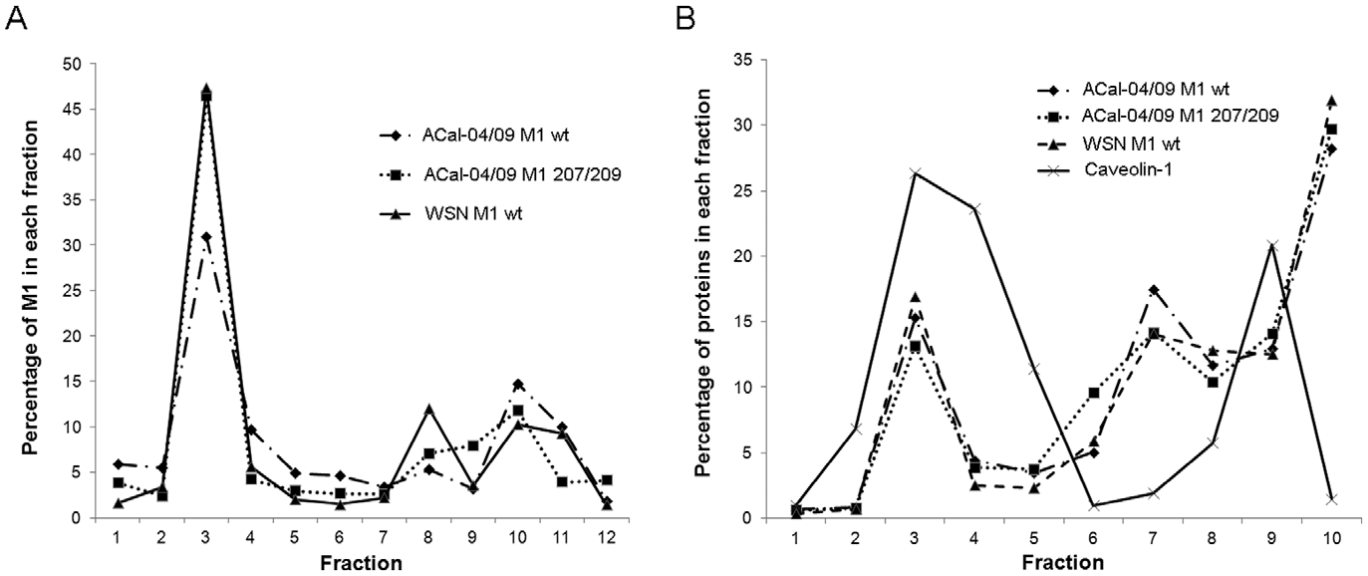
The pH1N1 M1 protein associates as efficiency with the plasma membrane and lipid raft domains as other influenza M1 proteins. 293 T cells were transfected with WT ACal-04/09 M1, ACal-04/09 M1 207/209 and WSN M1 protein expression plasmids. At 24 hpi, cells were (A) homogenized with a 27 gauge needle, mixed with 70% sucrose, and subject to membrane flotation analysis or (B) lysed in 0.5% Triton×100 and mixed with 70% sucrose for lipid raft isolation. Fractions were collected from the top of each gradient following ultracentrifugation, separated by gel electrophoresis and analyzed by Western Blot for M1 protein. Bands intensities were quantified using BioRad Software.

A recent study showed that increased membrane association of M1 fused with membrane targeting peptide enhanced production of VLP from transfected cells, suggesting that efficiency of plasma membrane association affect the VLP production [37]. To address whether ACal-04/09 M1 can more readily associate with the plasma membrane compared to other influenza M1 proteins, we performed membrane flotation analysis on 293T cells transfected with ACal-04/09 or WSN M1 proteins (Fig. 7A). In addition, we included ACal-04/09 M1 207/209 to determine if any observed differences were due to the pH1N1 specific residues. Our results, however, indicate no measurable difference in M1 association with the membrane when comparing either ACal-04/09 construct with WSN M1. All of them showed that 46 to 56% M1 association with membrane as determined by protein contents of fractions 2–4 (Fig. 7A).

During viral assembly at the plasma membrane, M1 and other viral proteins localize to lipid raft domains which are composed mainly of glycosphingolipids and are known to be resistant to extraction with ice-cold Triton X-100. To determine if WT ACal-04/09 M1 localizes more efficiently within the specialized lipid raft compartments than other influenza M1 proteins, we performed lipid raft isolation on 293T cells expressing ACal-04/09 M1, ACal-04/09 M1 207/209, or WSN M1 (Fig. 7B). Similar to the membrane flotation analysis, lipid raft isolation revealed no difference in M1 association between influenza strains. Thus, association of M1 with lipid membrane or raft compartment is unlikely to be the major factor that enhances VLP production by ACal-04/09 M1.

## Discussion

The pH1N1 strain emerged from swine in Mexico and rapidly spread to more than 200 countries [39]. This novel pandemic virus arose from a reassortment event between a North American TR swine virus and a Eurasian swine virus. Both TR and Eurasian swine viruses had previously caused sporadic infections in humans, but failed to spread from person-to-person [6], [7]. Therefore, it is likely that genes introduced by the reassortment and/or mutations which occurred after the reassortment caused emergence of the highly transmissible pH1N1 viruses. In fact, a recent study suggested the contribution of NA and M genes, which were derived from the Eurasian swine lineage, to virus transmissibility of pH1N1 in the ferret model [9]. For efficient transmission among human hosts, multiple factors are involved including efficient virus attachment to upper respiratory tract, replication in these tissues, and release and aerosolization of virus particles [40]. Aerosolized droplets range in size and thus the capacity to travel over long distances. Large droplets measuring greater than 6 µm can reach only several meters and may therefore contribute to transmission mainly by direct contact, whereas smaller droplets less than 1µm are carried much further and with greater propensity to transmit virus via inhalation [41]. Ferrets infected with the pH1N1 were found to release a higher number of submicron particles containing viral RNA and exhibited more efficient viral transmission than either TR or Eurasian swine viruses [9], suggesting a possible role for small aerosolized droplets in enhancing viral transmission. Moreover, these submicron droplets likely contain spherical rather than filamentous virions due to obvious size constraints, thereby strengthening the hypothesis that strains producing spherical virions have the potential to transmit more efficiently.

In this study, we determined the role of the pH1N1 M1 protein in virus morphology and growth *in vitro*. We report that the majority of purified pH1N1 viruses grown in A549 cells formed spherical virions. IF analysis of cells infected with pH1N1 revealed punctae structures at the cell surface, which clearly differ from the filamentous protrusions observed in TR virus-infected cells. Our data is in accord with the initial structural analysis performed by members of the CDC (http://www.cdc.gov/h1n1flu/images.htm), yet disagrees with an investigation which reported the pH1N1 strain as predominantly filamentous when analyzed using MDCK or primary human airway epithelial cells [28] or with a study reporting that 60% of purified A/California/07/2009 grown in MDCK cells was filamentous [9]. Possible explanations for these discrepancies include differences in passage history of the viruses and cell types used in these studies. To address the first issue, we infected cells with nasal swab samples from patients which had RT-PCR confirmed pH1N1 infection, and determined the cell surface structures by IF. These viruses showed punctae or very short filament protrusions from cell surfaces, similar to the ACal-04/09 strain (Figs. 1 and 3). Secondly, characterization of influenza in different cell types may yield contrasting results. However, in our cell cultures, virion morphology was found to be consistent between A549 cells and primary human lung epithelium. Although it is difficult to predict the virion morphology produced in the human respiratory tract, our data suggest that pH1N1 viruses contains a phenotype that readily produces spherical or very short filamentous virions from infected human respiratory epithelial cells.

By introducing TR M1 residues into pH1N1 M1, we identified three residues at 30, 207 and 209, which affected virion morphology and virus growth. Single mutations at these residues, reduced plaque size, growth rate and overall virus production (Fig. 5 and Table 3). pH1N1 mutants containing 207S or both 207S and 209A induced especially long filamentous protrusions at the cell surface, possibly reflecting inefficient fission and release of budding virions (Fig. 4). Sequence analysis revealed position 209 to be a highly conserved lineage-specific residue at which all Eurasian swine viruses between 2000 and 2008 possessed Thr, which differs from Ala found in the classical swine viruses (Table 2). Though residue 142 was also found to be lineage-specific, it did not appear to affect virion morphology by itself in our study. In contrast, Ser and Asn found at positions 30 and 207 in the pH1N1 M1 sequence rarely exist in other swine strains. Based on these data, it is possible that additional mutations at residue 30 and/or 207 introduced to the Eurasian M segment of the precursor reassortant virus contribute to the efficiency of spherical virion production and transmission of pH1N1.

It is unclear at this stage how pH1N1 M1 mutations at 30, 207 and 209 affect virus morphology. Influenza virus assembly and budding is a complex, multi-step process involving interactions between viral and cellular proteins. Many viral protein interactions are known to affect virus assembly and morphology, such as M1-HA, M1-NA, M1-M2, and M1-vRNP interactions [42]. The exact site of M1 interaction with HA and NA is not yet identified, however, crosslinking between the cytoplasmic tails of HA and NA with M1 is postulated. Viruses containing mutations in the cytoplasmic tails of both HA and NA have greatly altered morphology and, as seen with viruses harboring deletions in the M2 cytoplasmic tail, exhibit reduced incorporation of M1 and vRNP. This is suggestive that a structural change in M1 induced by interaction with the cytoplasmic tails of viral transmembrane proteins affects the interaction with vRNP and stabilizes the morphology of progeny virions [11], [43], [44]. However, based on our data, it is likely that pH1N1 M1 itself has a unique phenotype that facilitates virion production because of its ability to produce VLP efficiently unlike other virus strains tested (Fig. 6). Mutations at residues 30, 207 and 209, which resulted in enhanced formation of filamentous protrusion in infected cells, greatly reduced VLP production from transfected cells. In contrast, a mutation at residue 142 barely affected infected cell surface structure and did not reduce VLP production. A previous study suggested that an association of membrane targeting peptide to M1 increased VLP production in transfected cells, suggesting that plasma membrane association of M1 is critically important for VLP formation [37]. However, we did not detect any major difference in membrane association between ACal-04/09 M1, ACal-04/09 M1 207/209, and WSN M1 (Fig. 7A), indicating that efficient production of VLP by ACal-04/09 M1 is not due to enhanced membrane association. Additionally, we determined M1 association with lipid raft domains in the plasma membrane, which are considered to be the sites of influenza virus assembly and budding [45], [46]. However, we found similar levels of M1 co-localization with lipid raft domains following Triton×100 extraction between the viruses tested, suggesting comparable targeting to known sites of influenza virus assembly. Thus, the unique feature of the pH1N1 M1 protein that allows for efficient VLP production is unlikely to be due to its localization to budding sites at the plasma membrane.

Recent cryo-electron tomography data indicate that M1 forms a helical net under the viral membrane. The pitch of the helical turn of the M1 protein differs between spherical and filamentous virions, suggesting that M1-M1 interaction and its structure determine virion morphology [47]. A helix formation provides a structural mechanism by which assembly of M1 subunits at the plasma membrane drive budding. Therefore, M1-M1 interaction sites are likely to be the key determinant of virion morphology. Another possibility that alters virion morphology and VLP production could be interaction with cellular proteins that mediate pinch-off of budding virus or VLP. Although it has not been identified which cellular factors are responsible for the pinch off of budding influenza viruses, efficiency of the pinch off is likely to affect the morphology of the viruses. It is unclear if residues 30, 207 and 209 are involved in the specific helical contact sites in the matrix layer or interaction with cellular factors required for pinch off of budding virus. However, the unique phenotype of pH1N1 M1 in efficient VLP production by itself may reflect its ability to induce spherical virion formation and efficient transmission among human hosts.

## Materials and Methods

### Cells and Viruses

Human lung epithelial (A549), human embryonic kidney (293T), and Madin-Darby canine kidney (MDCK) cells were cultured in Dulbecco’s modified Eagle’s medium (DMEM) supplemented with 8% fetal calf serum (FCS). Porcine kidney (LLC-PK1) cells were obtained from American Type Culture Collection and were cultured in Medium 199 supplemented with 8% FCS. Normal human bronchial epithelial (NHBE) cells obtained from Lonza were maintained in bronchial epithelial cell basal medium (BEBM) with the supplements provided. Triple reassortant swine isolates A/Wisconsin/87/2005 (H1N1), A/Iowa/01/2006 (H1N1), A/Iowa/02/2006 (H1N1), A/Ohio/02/2007 (H1N1) and A/Minnesota/03/2008 (H1N1) [7] were obtained from A. Klimov (Centers for Disease Control and Prevention, Atlanta, GA). A/chicken/Nanchang/3-120/01, A/WSN/33 (H1N1), and A/Aichi/2/68 (H3N2) were described previously [48]. Nasal swab specimens from nine patients with PCR-confirmed pH1N1 infection were collected by J. Treanor (University of Rochester Medical Center, Rochester, NY). Collection of nasal swabs was approved by the Institutional Review Board of the University of Rochester, and all subjects gave written informed consent before participation.

### Plasmids

cDNAs encoding Nan, Aichi and ACal-04/09 M1 protein were synthesized by RT-PCR using SuperScript III One-step RT-PCR Platinum Taq HiFi (Invitrogen) from total RNAs extracted from virus-infected cells, and subcloned into pCAGGS. Mutations in the M1 gene were created using the QuikChange II site-directed mutagenesis kit (Agilent Technologies, Santa Clara, CA). cDNAs used to rescue ACal-04/09 were synthesized from RNAs provided by R. Webster and R. Webby (St. Jude Children’s Hospital, Memphis, TN) using RT-PCR kit and subcloned into the pPolI vector given to us by Y. Kawaoka (University of Wisconsin, Madison, WI). Amino acid substitutions at positions 30, 142, 207, and 209 were introduced into the pPolI-M plasmid by site-directed mutagenesis and used for rescue of the ACal-04/09 M1 mutant viruses.

### Virus Rescue

Viruses were rescued using the 12-plasmid rescue system developed by Neumann and Kawaoka [49]. Briefly, MDCK/293T co-cultures were transfected with pCAGGS-ACal-04/09-PB1, -PB2, -PA and -NP expression plasmids together with pPolI vectors encoding each of the gene segments using lipofectamine 2000 (Invitrogen). Plaque purified stocks were propagated in MDCK cells with DMEM containing 0.15% bovine serum albumin (BSA) and 2 ug/ml tosylsulfonyl phenylalanyl chloromethyl ketone (TPCK) treated trypsin. Mutations in the M segment were confirmed by sequence analysis, and viruses were titered in MDCK cells by immunofluorescence analysis of the nucleoprotein.

### IF and Electron Microscopy

A549 or LLC-PK1 cells were infected with the viruses at MOI 1 and incubated with DMEM containing 0.15% BSA for 18 h. For detection of viral surface proteins by IF, cells fixed with 4% paraformaldehyde were incubated with anti-H1N1 mouse serum supplied by D. Topham (University of Rochester Medical Center, Rochester, NY) followed by goat anti-mouse IgG conjugated with Texas Red (Invitrogen). Images were taken using an Olympus inverted microscope. For scanning electron microscopy, cells were fixed with 2.5% glutaraldehyde and visualized under a Zeiss Auriga supra 40VP Field Emmison microscope. For the analysis of virions, supernatant from virus-infected A549 cells was spun through a 100 K Amicon filter unit (Millipore) for 18 min at 3,500 rpm. A 10 µl volume of concentrated virus was absorbed to carbon-coated 200 square mesh nickel grids for 3 min, washed once with PBS(+), and negatively stained for 1 min with filter sterilized 2% phosphotungstic acid. Transmission electron micrographs were obtained at ×200,000.

### Viral Growth Analysis

MDCKs in 6-well culture plates were infected with the viruses at MOI 0.01 and incubated in 2 ml DMEM containing 0.15% BSA and 2 ug/ml TPCK-treated trypsin. At various times points, 200 µl of the culture supernatant was harvested and replaced with an equal volume of fresh media. Virus titers were determined in MDCK cells as described by Reed and Muench [44]. For plaque analysis, MDCK cells infected with WT or mutant ACal-04/09 viruses were incubated at 37°C for 4 days. Cells were fixed for 10 min in 10% trichloroacetic acid (TCA). After removal of overlay medium, cells were stained with 0.1% crystal violet in 20% ethanol.

#### Virus-like particle production

293T cells transfected with pCAGGS-M1 vectors were labeled for 24 h with ^35^S-Met/Cys in DMEM lacking methionine and cystine. Cell culture supernatants were cleared of cell debris by low speed centrifugation, layered onto 20% sucrose cushions and centrifuged at 35,000 rpm for 2 h in an SW41 rotor (Beckman). Pellets containing virus-like particles were resuspended in laemmli buffer (BioRad) and analyzed by SDS-PAGE. Cell lysates were harvested in RIPA buffer (50 mM Tris, pH 7.4, 150 mM NaCl, 2 mM EDTA, 0.1% SDS, 1% Triton X-100), clarified, and separated by SDS-PAGE. Proteins were transferred to a PVDF membrane and subject to Western Blot analysis with anti-M1 GA2B monoclonal antibody (Sigma). Band intensities were quantified with BioRad Quantity One software and the amount of M1 detected in culture supernatant was normalized to M1 expressed in cell lysates.

### Membrane Flotation Analysis

Flotation analysis on 293 T cells transfected with pCAGGS-M1 plasmids was performed as described by Sanderson et al. [45] in a 6-well format. Briefly, cells were washed 24 h post-transfection with cold PBS(+), harvested by scraping and pelleted by low speed centrifugation. Following resuspension in 0.7 ml hypotonic lysis buffer (10 mM Tris (pH 7.5); 10 mM KCl; 5 mM MgCl2), cell lysates were incubated on ice for 10 min, passaged 15 times through a 26 gauge hypodermic needle and centrifuged 7 min at 1,000×*g*. Half ml aliquots of supernatant were mixed with 3.5 ml 70% sucrose in low salt buffer (LSB) (50 mM Tris HCl (pH 7.5) 25 mM KCl and 5 mM MgCl2), overlaid with 5.5 ml 55% sucrose and 2 ml 10% sucrose in LSB and ultracentrifuged at 38,000 rpm for 18 h at 4°C in an SW41 rotor. One-ml fractions were collected from the top of each gradient to which 0.25 ml 100% trichloroacetic acid was added for protein precipitation. After 10 min incubation on ice, fractions were spun at top speed for 5 min. Protein pellets were then washed twice with cold acetone, heated to 95°C for 5 min and resuspended in NuPAGE sample buffer (Invitrogen) for SDS-PAGE and Western Blot analysis with anti-M1 GA2B antibody. Band intensities within each fraction were quantified using BioRad Quantity One software.

### Lipid Raft Isolation

Isolation of lipid rafts from 293 T cells transfected with pCAGGS-M1 plasmids was performed as described by Carrasco et al. [46] with several modifications. Cells were washed 24 h post-transfection with cold PBS(+) and incubated on ice for 20 min in 0.5 ml LSB containing 0.5% Triton×100. Each cell lysate (0.3 ml) was mixed with 0.7 ml 70% sucrose in LSB and overlaid with 2 ml 30% sucrose and 1 ml 2.5% sucrose in LSB. Gradients were spun at 28,000 rpm for 16 h at 4°C in an SW55 rotor, after which 0.4 ml fractions were collected from the top. Protein was precipitated with TCA as described above, resuspended in NuPAGE sample buffer, separated in a 4–12% Bis-Tris gel (Novex), and subject to Western Blot analysis with anti-M1 GA2B. Anti-caveolin 1 polyclonal antibody (abcam) was used as a marker for fractions containing lipid raft associated proteins. Band intensities within each fraction were quantified using BioRad Quantity One software.
